# The Impact of Clinical Supervision on the Mental Health Nursing Workforce: A Scoping Review

**DOI:** 10.1111/inm.13463

**Published:** 2024-10-27

**Authors:** Joshua McDonough, Kate Rhodes, Nicholas Procter

**Affiliations:** ^1^ Mental Health and Suicide Prevention Research and Education Group Clinical and Health Sciences, University of South Australia Adelaide South Australia Australia

**Keywords:** clinical supervision, health workforce, mental health nursing, scoping review

## Abstract

Clinical supervision is a psychologically safe practice that aims to provide support and training for workers within the healthcare industry, including mental health nurses. Over the years, clinical supervision has been cited as a practice to improve workforce outcomes for both individual mental health nurses and the organisations they work in. The aim of this scoping review is to examine the evidence exploring the relationship between clinical supervision and workforce outcomes for mental health nurses. Twenty‐eight articles sourced from six databases were included in this study. The most frequently evaluated workforce outcomes were competence (*n* = 14), workplace culture (*n* = 13) and compassion (*n* = 7). Studies reported that the association between clinical supervision and workforce outcomes was predominantly positive, but there were mixed results for competence, workplace culture, job satisfaction and burnout. Details on the type of clinical supervision received by participants were limited, and most of the evidence included in this review included qualitative research and participants self‐reporting their perceived benefits from clinical supervision, as opposed to using validated instruments in experimental and/or longitudinal study designs. Organisations should be hesitant to implement mandatory clinical supervision within workplaces, as this could have the opposite effect on workforce outcomes for mental health nurses who are already time‐poor and overworked, as well as those who are indifferent or hostile to clinical supervision.

## 1 Introduction

Mental health nurses are a crucial element of multidisciplinary mental health care in Australia. Their role is to coordinate therapeutic and recovery‐oriented practices with other care providers to reduce psychological harm for clients, including those experiencing suicidal distress (Santangelo, Procter, and Fassett 2018). Mental health nurses work in emotionally and occupationally challenging environments, and as a result experience high rates of burnout, fear, guilt and self‐doubt (Cranage and Foster 2022). These stresses lead to difficulties in attracting and maintaining specialised mental health nurses in the workforce. In 2023 the Australian Department of Health and Aged Care reported that there was a 32% shortfall in mental health workers compared to the target, including mental health nurses, and this gap is predicted to widen based on current trends (The Department of Health and Aged Care 2023). Staff shortages lead to poor workforce outcomes at an individual and a systems level. This can include chronically understaffed services, poor staff well‐being and burnout, lower workplace safety and worse outcomes for clients receiving care (Andel et al. 2022; Thompson, Senek, and Ryan 2024). As a result of these challenges, the National Mental Health Workforce Strategy outlines the goals of attracting, training, maximising, supporting and retaining workforces within the mental health care space to build sustainability (The Department of Health and Aged Care 2023).

Clinical supervision is a formalised approach to professional development that creates a supportive space for healthcare workers to critically reflect on experiences relating to their work. These experiences can include professional and clinical development, boundaries, caseload management and clinical decision‐making and practice, among others (Thomas and Isobel 2019). The relationship begins with a mutually supported formal agreement between a supervisor and either one (individual supervision) or multiple (group supervision) supervisees. Clinical supervision typically involves regular structured meetings in safe and private settings (Fowler and Cutcliffe 2010). Clinical supervision is used in several healthcare professions, including nursing, medicine, psychology and allied health.

Systematic clinical supervision for healthcare workers has been reported to show benefits for people providing and receiving healthcare. These include supporting staff who work in isolation, resolving interpersonal workplace issues, facilitating deeper knowledge and competencies of contemporary practice and reducing staff fatigue and burnout (Marshman, Hansen, and Munro 2022; Dickinson and Wright 2008). Whilst challenging to evaluate, reported client benefits are better relationships with their providers and increased perceptions of care quality (Bambling et al. 2006; White and Winstanley 2010). Given these benefits, clinical supervision has been put forward as a method to support the mental health nursing workforce with the goal of attracting, training, supporting and retaining mental health nurses.

Defining and implementing clinical supervision within healthcare settings has had difficulties over time. Clinical supervision does not have a universally agreed‐upon definition. In the past, ‘clinical supervision’ has been interpreted in different ways, for example, as performance management through clinical‐based observation (Sharrock et al. 2019). As a result, there can be reluctance among healthcare workers when discussing clinical supervision within a workplace, as some workers see it as aiming to punish or criticise workers. While the aim of clinical supervision is to provide a supportive and nurturing environment, there can be adverse outcomes for workers who receive poor‐quality clinical supervision. Due to the sensitive and personal nature of issues discussed in clinical supervision, examples, where there have been breaches in confidentiality of supervision, can result in shame, doubt in their ability to perform their role and mistrust of others in the workplace (Ellis et al. 2014). This can have negative outcomes for individuals and workplace organisations.

There are criticisms of the research that has aimed to evaluate the effectiveness of clinical supervision, as there are often multiple confounding factors when conducting research within a hospital setting. (Dilworth et al. 2013; Carpenter, Webb, and Bostock 2013). Previous literature reviews collating the evidence evaluating clinical supervision effectiveness include focusing on workforce outcomes in healthcare workers broadly (Martin et al. 2021), and the association between different approaches to providing supervision and formative and restorative outcomes of supervisees (Bradley and Becker 2021). There has not been a review of the impacts of clinical supervision on workforce outcomes with a focus on mental health nurses. This review is needed because mental health nursing has the most personnel with mental healthcare workers globally and experience difficulties in implementing systematic clinical supervision within services partly because their role involves shift work to ensure clients receive 24 h care (Stewart et al. 2022).

### 1.1 Aims and Objectives

The aims of this scoping review are:

- Identify, map and analyse the published evidence evaluating the effect of clinical supervision on workforce outcomes (both personal and system/structural) specific to mental health nursing,
- Identify gaps in evidence for the evaluation of clinical supervision on individual and system workplace outcomes.

## 2 Methods

This scoping review has followed the Joanna Briggs Institute guidance for scoping reviews (Peters et al. 2020). A protocol for this scoping review has been published elsewhere which outlines the theoretical underpinning of the concepts explored (Mcdonough, Rhodes, and Procter 2024).

### 2.1 Search Strategy

The search strategy for this review was completed with the support of a senior University Clinical and Health Sciences Librarian. Following a population concept context (PCC) framework (Pollock et al. 2023), we determined the population to be mental health nurses, the concept to be clinical supervision and the context to be workforce attributes. The databases searched in this review were Medline, CINAHL, PsycINFO and Embase because they are the most relevant to the review framework (Bramer et al. 2017). The initial search was developed for Medline (See Appendix 1) and adapted for subsequent databases. Google searches and the website, Grey Matters, were used to identify relevant grey literature. All included articles underwent backward citation searching (where the reference list of an included article was screened) and forward citation searching (where the list of articles that had cited an included article was screened) to ensure completeness of the screening process. The final search was conducted on 6/10/2023, with no date limits applied.

### 2.2 Eligibility Criteria

Articles needed to satisfy the following inclusion criteria, and none of the exclusion criteria to be included in this review:

Inclusion criteria:

1. Primary research with mental health nurses as participants, and
2. Evaluating the effect of clinical supervision in relation to individual and systems‐level workforce outcomes

Exclusion criteria:

1. Secondary or non‐research articles, (including research protocols, commentaries, editorials, conference papers, among others)
2. Published in languages other than English.
3. Studies involving students or student‐based mentorship structures (e.g. preceptorship).

### 2.3 Rationale

A focus on primary studies of mental health nurses was decided to ensure the results of this review were directly relevant to the mental health nursing workforce and reduced the possibility of repeating data or combining it with other professions.

### 2.4 Process

Screening for this review involved two steps after duplicate articles had been removed. First, two authors (J.M., K.R.) used the inclusion and exclusion criteria to separately screen identified articles by title and abstract using the online‐based computer software, Covidence (Veritas Health Innovation, Melbourne, Australia). Articles not excluded were then screened by full text. Disagreements regarding the relevance of an article between authors were discussed to resolve conflicts. Conflicts that could not be resolved were mediated by a third author (N.P.).

### 2.5 Data Extraction

Predefined data items were extracted by one author (J.M.), using a custom‐designed Excel spreadsheet, then extracted and cross‐checked by another (K.R.). These items included: authorship and year published, country of origin, study design, clinical setting, sample size, participant age and sex, model of supervision participants received, type of supervision undertaken (individual or group), length and frequency of supervision, workforce outcome(s) measured (personal attributes and system/structural attributes) and research instruments used (e.g. the Manchester Clinical Supervision Scale).

### 2.6 Data Analysis

Data analysis for workforce outcomes followed a narrative synthesis approach (Popay et al. 2006). This was done using the defined workforce outcomes described in the scoping review protocol (Mcdonough, Rhodes, and Procter 2024) to code synthesise the data using NVivo 14 software (QSR International, Chadstone, Victoria, Australia). Analysis was performed by one author (J.M.), with coding accuracy checked by a second author (K.R.).

## 3 Results

### 3.1 Study Characteristics

**FIGURE 1:**
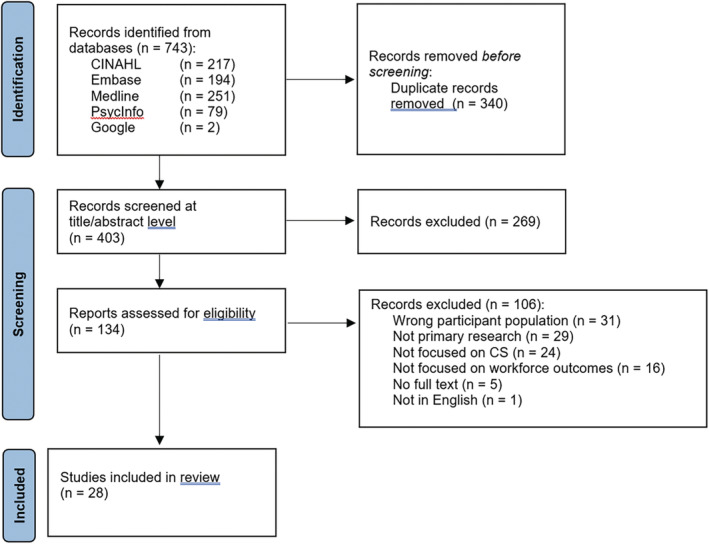
PRISMA diagram mapping the process of article screening.

The search yielded a total of 743 articles and 401 after duplicates were removed, with 28 articles included in the review. Please see Figure 1 for the PRISMA diagram summarising the process.

### 3.2 Characteristics of Sources of Evidence

Of the 28 included articles, most studies were from the United Kingdom (*n* = 13), followed by Australia (*n* = 5) and Sweden (*n* = 3). The most common methodology was qualitative (*n* = 12), with nine quantitative studies, and seven using a mixed‐methods approach. All but one of the included studies were sourced from peer‐reviewed journals, with one thesis also included. See Table 1 for full study characteristics.

**TABLE 1:** Summary characteristics of the included studies.

Study details		Participant details							
Author (Year)	Study design	Country	Population description	Clinical setting (Outpatient, inpatient, both)	Sample size (*n*)	Age (*M* [SD], range=)^a^	Sex		Response Rate
							F	M	
Arvidsson, Löfgren, and Fridlund (2001)	Qualitative interviews	Sweden	Psychiatric nurses	Both	10	42, 26–55	60%	40%	Not reported
Carney (2005)	Cross‐sectional survey	England, UK	Mental health nurses	In‐patient	35	Not reported	Not reported	Not reported	70%
Cleary and Freeman (2005)	Ethnographic; Observational fieldwork study and interviews	Australia	Mental health nurses	In‐patient	10	Not reported	Not reported	Not reported	Not reported
Cutcliffe, and Burns, J. (1998)	Case study	England	Psychiatric nurses	In‐patient	3	Not reported	Not reported	Not reported	N/A
Delgado et al. (2020)	Cross‐sectional survey	Australia	Mental health nurses	Both	482	44.6 (12.32)	80%	20%	Not reported
Edwards et al. (2006)	Cross sectional survey	Wales	Mental health nurses	Community	260	42, 25–64	62%	38%	32%
Feerick, Doyle, and Keogh (2021)	Qualitative interviews	Ireland	Forensic mental health nurses	In‐patient	10	35	60%	40%	5.50%
Gonge and Buus (2011)	Cross‐sectional questionnaire survey	Denmark	Psychiatric nurses, auxiliary nurses, OTs and social educators	Both	136	46.3, 22–65	85%	15%	60.70%
Hallberg (1994)	Cohort study, mixed methods	Sweden	Mental health nurses	In‐patient	227	38.6 (8.9)	64%	36%	80%
Hamilton et al. (2023)	Mixed methods	Australia	Mental health nurses	In‐patient	67	35.98 (11.9)	75%	25%	52%
Hercelinskyj (2010)	Qualitative interviews	Australia	Mental health nurses, nursing students	Both	11	Not reported	Not reported	Not reported	Not reported
Ho (2007)	Qualitative focus groups	UK	Mental health nurses	In‐patient	6	Not reported	Not reported	Not reporter	Not reported
Hughes and Morcom (1998)	Longitudinal quantitative survey (self‐report on paper)	UK	Mental health nurses	In‐patient	Not reported	Not reported	Not reported	Not reported	50%
Hyrkas (2005)	Cohort quantitative survey (self‐report)	Finland	Mental health nurses	In‐patient	569	41.8, 23–60	78%	22%	Not reported
Kelly, Long, and Mckenna (2001)	Cohort quantitative survey (self‐report)	UK	Mental health nurses	Community	153	21–60	36%	64%	61.20%
Lakeman and Glasgow (2009)	Action research (qualitative)	Trinidad & Tobago	Mental health nurses and enrolled nurses	In‐patient	10	43	100%	0%	Not reported
Lee and Kim (2022)	Qualitative interviews	South Korea	Psychiatric mental health nurses	Both	19	40–65	100%	0%	Not reported
Long et al. (2014)	Cohort quantitative survey (self‐report)	UK	Mental health nurses and health care assistants	In‐patient	128	Not reported	Not reported	Not reporter	67%
Maclaren, Stenhouse, and Ritchie (2016)	Qualitative interviews	UK	Mental health nurses	Not reported	811	Not reported	86%	14%	Not reported
Mccarron, Eade, and Delmage (2018)	Longitudinal Mixed‐methods surveys (six open‐ended qual questions) pre and post‐differences statistically analysed	UK	Mental health nurses and health care assistants	In‐patient	69	Not reported	Not reported	Not reported	Pre: 29% Post 36%
McKenna et al. (2010)	Mixed methods survey	New Zealand	Mental health and addiction nurses	Both	73	Predominantly (74%) middle‐aged.	73%	27%	60%
Oates (2018)	Mixed methods survey and interviews	UK	Mental health nurses	Not reported	237 surveys; 27 interviews	Not reported	Survey: 72% Interviews: 82%	Survey: 28% Interviews: 18%	Not reported
Odeyemi, Morrissey, and Donohue (2018)	Qualitative interviews	Ireland	Mental health nurses	Community	8	Not reported	75%	2%	100%
Olofsson (2005)	Qualitative interviews	Sweden	Psychiatric nurses	In‐patient	21	47, 27–63	Not reported	Not reported	92%
Robinson, Murrells, and Smith (2005)	Cohort, longitudinal mixed methods survey questionnaire and interviews	UK	Mental health nurses	In‐patient	444	Not reported	70%	30%	Baseline: 82% at Follow‐up: 80%
Scanlon and Weir (1997)	Qualitative interviews	UK	Mental health nurses	Both	10	Not reported	60%	40%	Not reported
Sherring and Knight (2009)	Cohort quantitative survey (self‐report)	UK	Mental health nurses	Not reported	166	< 25–> 66	73%	27%	35%
White and Winstanley (2010)	Longitudinal mixed methods RCT	Australia	Mental health nurses, clients and unit staff	Both	186	Not reported	Not reported	Not reported	Not reported

^a^ Reported as best as possible with information available in the paper.

### 3.3 Description of Clinical Supervision

Reporting on the specifics of the clinical supervision received by participants was limited. Most (*n* = 17) of the included studies did not report on the model of clinical supervision used by participants. Among those that did, Procter's Model was the most frequently cited (*n* = 5). Group supervision was the most reported mode of clinical supervision (*n* = 7), with four studies including both group and individual supervision, and zero evaluating individual supervision only. The remaining 17 studies did not report on the mode of supervision. Considering workforce outcomes, 14 included studies reported on both individual‐ and system‐level attributes, 12 individual attributes only and two system attributes only. See Table 2 for full details of clinical supervision evaluation in the included studies.

**TABLE 2:** Summary of the clinical supervision received by participants in the included studies and workforce outcomes measured.

Study details	Evaluation details				
Author (Year)	Model(s) of CS used	CS Modality (Individual, Group, Mixed	Length and frequency of CS	Workforce outcomes measured (measurement instrument)	
				Individual	Systems
Arvidsson, Löfgren, and Fridlund (2001)	Sarvimaki and Stenbock‐Hult (1993) nursing model; and Johns (1993) model of structured reflection	Group	2 h, fortnightly	Self‐confidence (qualitatively) Competence (qualitatively) Knowledge (qualitatively) Job satisfaction (qualitatively)	Morale (qualitatively)
Carney (2005)	Procter's model	Not reported	Not reported	Competence (study‐specific instrument)	Morale (study‐specific instrument)
Cleary and Freeman (2005)	Not reported	Mixed	Not reported	Job satisfaction (Observation and qualitatively) Self‐confidence (Observation and qualitatively) Competence (Observation and qualitatively)	Organisational support (Observation and qualitatively) Workplace culture (Observation and qualitatively)
Cutcliffe, and burns, J. (1998)	Procter's model	Not reported	Not reported	Competence (Case study observation)	None
Delgado et al. (2020)	Not reported	Not reported	Not reported	Resilience (Resilience at work scale)	None
Edwards et al. (2006)	Not reported	Mixed	Varied	Burnout (Maslach burnout inventory)	None
Feerick, Doyle, and Keogh (2021)	Not reported	Not reported	Not reported	Burnout (qualitatively)	None
Gonge and Buus (2011)	Reflexive practice, psychodynamic framework	Group	Varied	Job satisfaction (The Copenhagen Psychosocial Questionnaire) Burnout (Maslach burnout inventory/The Copenhagen Psychosocial Questionnaire)	None
Hallberg (1994)	Psychodynamic framework	Group	2 h, every 3 weeks	Professional identity (qualitative) Burnout (Maslach Burnout Inventory) Compassion (qualitative) Self‐confidence (qualitative) Competence (qualitative)	Workplace culture (qualitative) Morale (qualitative)
Hamilton et al. (2023)	Procter's model	Not reported	Varied	Knowledge (Manchester Clinical Supervision Scale) Compassion (qualitative)	Workplace culture (qualitative) Organisational support (qualitative)
(Hercelinskyj 2010)	Not reported	Not reported	Not reported	Professional identity (qualitative)	Workplace culture (qualitative)
Ho (2007)	Psychoanalytical framework	Group	Not reported, Every 2–4 weeks	Competence (qualitative)	None
Hughes and Morcom (1998)	Not reported	Not reported	Not reported, Every 2–6 weeks	Job satisfaction (study‐specific instrument) Self‐confidence (study‐specific instrument) Self‐efficacy (study‐specific instrument) Competence (study‐specific instrument)	Workplace culture (study‐specific instrument)
Hyrkas (2005)	Not reported	Mixed	Varied (< 45 min– > 2 h), Varied (Every 2 weeks‐fewer than monthly)	Job Satisfaction (Minnesota Job Satisfaction Scale)	None
Kelly, Long, and Mckenna (2001)	Not reported	Not reported	Not reported	Competence (Study‐specific instrument) Self‐confidence (Study‐specific instrument)	Workplace culture (Study‐specific instrument)
Lakeman and Glasgow (2009)	Heron's peer supervision	Group	1–2 h, every 2 weeks	Competence (qualitative) Job satisfaction (qualitative) Compassion (qualitative)	None
Lee and Kim (2022)	Not reported	Not reported	Not reported	Compassion (qualitative) Competence (qualitative)	None
Long et al. (2014)	Mixed; Proctor's model	Not reported	Not reported, monthly or less than monthly	Resilience (Bradfield Supervision Scale) Competence (Bradfield Supervision Scale)	Workplace culture (Bradfield Supervision Scale)
Maclaren, Stenhouse, and Ritchie (2016)	Not reported	Not reported	Not reported	Compassion (qualitative)	Workplace culture (qualitative)
Mccarron, Eade, and Delmage (2018)	Not reported	Not reported	Not reported, varied	Burnout (qualitative) Competence (qualitative)	Workplace culture (qualitative)
McKenna et al. (2010)	TAPES model; Procter's model	Not reported	Not reported	None	Workplace culture (qualitative)
Oates (2018)	Not reported	Not reported	Not reported	Resilience (qualitative)	Workplace culture (qualitative)
Odeyemi, Morrissey, and Donohue (2018)	Not reported	Not reported	Not reported	Competence (qualitative)	Workplace culture (qualitative)
Olofsson (2005)	Reflective practice model	Group	2 h, ~every 3 weeks	Competence (qualitative) Compassion (qualitative)	Workplace culture (qualitative) Organisational support (qualitative)
Robinson, Murrells, and Smith (2005)	Not reported	Not reported	Not reported	None	Staff retention (study‐specific instrument)
Scanlon and Weir (1997)	Not reported	Not reported	Not reported	Compassion (qualitative) Self‐confidence (qualitative)	None
Sherring and Knight (2009)	Not reported	Mixed	Not reported, varied	Burnout (Maslach Burnout Inventory)	None
White and Winstanley (2010)	Not reported	Group	Not reported	Burnout (Maslach Burnout Inventory)	None

### 3.4 Workforce Outcome Measures

The most reported workforce outcome was staff competence, with no studies reporting on sick leave, organisational support or allowing time for clinical supervision (see Table 3).

**TABLE 3:** Number of times each workforce outcome was reported by the included studies.

Concept	Individual or system attributes	*n*
Competence	Individual	14
Workplace culture	System	13
Compassion	Individual	7
Job satisfaction	Individual	6
Self‐confidence	Individual	6
Burnout	Individual	6
Resilience	Individual	3
Support	System	3
Morale	System	3
Knowledge	Individual	2
Professional identity	Individual	2
Self‐efficacy	Individual	1
Staff retention	System	1
Sick leave	Individual	0
Time for clinical supervision	System	0
Organisational acceptance	System	0

#### 3.4.1 Competence

Fourteen of the included studies reported on the effect of clinical supervision on the personal attribute staff competence (Arvidsson, Löfgren, and Fridlund 2001; Carney 2005; Cleary and Freeman 2005; Cutcliffe, and burns, J. 1998; Hallberg 1994; Ho 2007; Hughes and Morcom 1998; Kelly, Long, and Mckenna 2001; Lakeman and Glasgow 2009; Lee and Kim 2022; Long et al. 2014; Mccarron, Eade, and Delmage 2018; Odeyemi, Morrissey, and Donohue 2018; Olofsson 2005). When qualitatively explored, included studies reported themes of identifying new approaches to care (Hughes and Morcom 1998; Lee and Kim 2022; Olofsson 2005), facilitating individual growth and recognising gains in experience (Cleary and Freeman 2005; Hallberg 1994), developing coping mechanisms (Cutcliffe, and burns 1998), enhancing individual and team practice (Ho 2007; Hughes and Morcom 1998; Lee and Kim 2022; Lakeman and Glasgow 2009), translating theory into practice (Lee and Kim 2022; Arvidsson, Löfgren, and Fridlund 2001) and managing workloads (Odeyemi, Morrissey, and Donohue 2018).

Carney (2005) reported that among their study sample of nurses who did not participate in clinical supervision, 71% suggested that clinical supervision did not help them in their clinical work. Conversely, Long et al. (2014) reported that mental health nurses receiving clinical supervision were statistically more likely than not to report benefits of change in practice, increased creativity and ability to discuss difficult practice issues. In a survey of community mental health nurses, 75% of responders reported that supervision improves the standard of care and 87% that new skills can be gained in supervision (Kelly, Long, and Mckenna 2001).

#### 3.4.2 Workplace Culture

Thirteen of the included studies reported on the effect of clinical supervision on workplace culture (Cleary and Freeman 2005; Hallberg 1994; Hamilton et al. 2023; Hughes and Morcom 1998; Kelly, Long, and Mckenna 2001; Long et al. 2014; Maclaren, Stenhouse, and Ritchie 2016; Mccarron, Eade, and Delmage 2018; Mckenna et al. 2010; Oates 2018; Odeyemi, Morrissey, and Donohue 2018; Olofsson 2005). These studies reported themes of improved teamwork and understanding (Hamilton et al. 2023; Hallberg 1994), reduced feelings of isolation within a workplace (Hughes and Morcom 1998; Kelly, Long, and Mckenna 2001) and greater connection between staff members (Oates 2018; Olofsson 2005; Hercelinskyj 2010). Additionally, Cleary and Freeman (2005) reported that group clinical supervision was able to be viewed positively due to the strong team culture of support, which in turn was strengthened by ongoing group clinical supervision by spending time with colleagues of different experience levels away from care settings. Kelly, Long, and Mckenna (2001) reported that 83% of participants believed that clinical supervision was essential for reducing isolation, and Long et al. (2014) reported that participants felt more supported by clinical supervision and less isolated as a result of participating in clinical supervision.

Some of the included studies reported on the negative impacts of poor clinical supervision, including negative impacts on ward culture (Mccarron, Eade, and Delmage 2018); psychologically unsafe environments for supervisees (Maclaren, Stenhouse, and Ritchie 2016); and ambivalence towards providing and receiving mandatory supervision when either supervisor or supervisee was resentful towards engaging with clinical supervision (Mckenna et al. 2010). Finally, Odeyemi, Morrissey, and Donohue (2018) reported that adding the responsibilities of clinical supervision to the staff's workload without addressing workload demand could be seen as overwhelming.

#### 3.4.3 Compassion

Seven of the included studies reported on the effect of clinical supervision on mental health nurses' compassion for clients and/or colleagues (Hallberg 1994; Hamilton et al. 2023; Lakeman and Glasgow 2009; Lee and Kim 2022; Maclaren, Stenhouse, and Ritchie 2016; Olofsson 2005; Scanlon and Weir 1997). All seven studies qualitatively reported that clinical supervision improved their compassion within the workplace. This was shown by enhancing their ability to meaningfully understand the people in their care (Lee and Kim 2022; Hallberg 1994; Lakeman and Glasgow 2009), understanding the actions of their colleagues (Scanlon and Weir 1997; Hallberg 1994), discussing and venting emotional triggers (Maclaren, Stenhouse, and Ritchie 2016) and the facilitation of new models of care (Olofsson 2005; Hamilton et al. 2023).

#### 3.4.4 Job Satisfaction

Six of the included studies reported the effect of clinical supervision on job satisfaction (Arvidsson, Löfgren, and Fridlund 2001; Cleary and Freeman 2005; Gonge and Buus 2011; Hughes and Morcom 1998; Hyrkas 2005; Lakeman and Glasgow 2009). Studies mentioning job satisfaction qualitatively noted that clinical supervision improved feelings of being reassured in their work roles (Arvidsson, Löfgren, and Fridlund 2001), viewing personal growth as a rewarding part of the job (Cleary and Freeman 2005; Lakeman and Glasgow 2009) and increasing motivation (Hughes and Morcom 1998). While Hyrkas (2005) reported no statistically significant association between receiving clinical supervision and job satisfaction (measured using the Managerial Job Satisfaction Scale 2018); however, they did report that people who provided clinical supervision to others had high levels of job satisfaction. Gonge and Buus (2011) reported that effective clinical supervision was significantly statistically correlated with job satisfaction (*p* = 0.03) using multivariate analysis.

#### 3.4.5 Self‐Confidence

Six of the included studies reported on the effect of clinical supervision on the self‐efficacy of mental health nurses (Arvidsson, Löfgren, and Fridlund 2001; Cleary and Freeman 2005; Hallberg 1994; Hughes and Morcom 1998; Kelly, Long, and Mckenna 2001; Scanlon and Weir 1997). All studies that qualitatively assessed self‐confidence reported positive associations with clinical supervision from participants (Arvidsson, Löfgren, and Fridlund 2001; Cleary and Freeman 2005; Hughes and Morcom 1998; Scanlon and Weir 1997; Hallberg 1994). Kelly, Long, and Mckenna (2001) reported that from a survey of community mental health nurses, 73.2% supported the idea that greater confidence was an effect of clinical supervision. This belief was stronger among participants who were engaging with clinical supervision, compared with those who were not.

#### 3.4.6 Burnout

Six of the included studies reported on the effect of clinical supervision on burnout (Edwards et al. 2006; Feerick, Doyle, and Keogh 2021; Gonge and Buus 2011; Hallberg 1994; Mccarron, Eade, and Delmage 2018; Sherring and Knight 2009). Feerick, Doyle, and Keogh (2021) reported a belief that clinical supervision would reduce the levels of burnout among forensic mental health nurses, referring to the nature of their working environments. Mccarron, Eade, and Delmage (2018) reported that among mental health nurses who self‐identified as receiving inadequate clinical supervision, 40% of participants in 2013 believed that this had a negative effect on personal stress and burnout, while the figure in 2016 was 20% of responders. Sherring and Knight (2009) and Edwards et al. (2006) using the Maslach Burnout Inventory (1997) reported statistically significant lower levels of burnout relating to emotional exhaustion in mental health nurses who had more frequent and effective clinical supervision. Gonge and Buus (2011) reported that effective clinical supervision was significantly correlated to less stress (*p* = −0.03) and more vitality (*p* = −0.04) using multivariate analysis. Finally, Hallberg (1994) reported that clinical supervision had no statistically significant longitudinal effect on burnout over time.

#### 3.4.7 Resilience

Three of the included studies reported on the effect of clinical supervision on staff resilience (Delgado et al. 2020; Long et al. 2014; Oates 2018). Oates (2018) and Long et al. (2014) qualitatively reported that mental health nurses viewed clinical supervision as a tool to manage the vicarious trauma experienced as part of the role, cope with difficult situations and was seen as vital to the social well‐being of participants at work. Delgado et al. (2020) used the resilience at work scale to show that clinical supervision had a statistically significant positive relationship with participants' resilience at work.

#### 3.4.8 Support

Three of the included studies reported on the effect of support for staff receiving or not receiving clinical supervision (Cleary and Freeman 2005; Hamilton et al. 2023; Olofsson 2005). Hamilton et al. (2023) suggested that while participants themselves valued clinical supervision, they faced barriers in organisational structures in completing it. Cleary and Freeman (2005) reported that although participants' employers had a policy of offering clinical supervision, participants were not able to access it due to staffing and time constraints, noting that they would prefer to have an option outside of scheduled hours that they could claim as time in lieu. Olofsson (2005) reported that participants appreciated the opportunity for reflexive practice and thinking that group supervision provided, which they would otherwise not experience in the workplace.

#### 3.4.9 Morale

Three of the included studies reported on the effect of clinical supervision on workplace morale (Arvidsson, Löfgren, and Fridlund 2001; Carney 2005; Hallberg 1994). Qualitatively studies reported that participants who were involved in group clinical supervision felt a sense of trust and shared responsibility that came from a better understanding of colleagues' way of working, creating a sense of solidarity, as well as creating spaces for difficult discussions of how and why they perform their work (Arvidsson, Löfgren, and Fridlund 2001; Hallberg 1994). Quantitatively, while nearly 50% of responders said that clinical supervision helped them feel ‘slightly’ more motivated, 25% responded their motivation had not changed.

#### 3.4.10 Knowledge

Two of the included studies reported on the effect of clinical supervision on staff knowledge (Arvidsson, Löfgren, and Fridlund 2001; Hamilton et al. 2023). Arvidsson, Löfgren, and Fridlund (2001) qualitatively reported that group clinical supervision was a useful tool for mental health nurses to continue gaining knowledge of clinical practice through gaining insight, handling terminology and understanding the essence of nursing theories. Hamilton et al. (2023) reported that mental health nurses implementing a Safewards Model of Care found clinical supervision was effective in developing mental health nurses' skills and knowledge through reflective practice.

#### 3.4.11 Professional Identity

Two studies reported on the effect of clinical supervision on an individual's professional identity (Hallberg 1994; Hercelinskyj 2010). Mental health nurses qualitatively reported that clinical supervision helped them have an increased awareness of their professional roles and identity (Hallberg 1994), and improve their ability to differentiate between their workplace and their professional issues, creating effective ways to manage workplace problems without taking them home (Hercelinskyj 2010).

#### 3.4.12 Self‐Efficacy

One included study reported on the effect of clinical supervision on staff's self‐efficacy (Hughes and Morcom 1998). Hughes and Morcom (1998) reported that participants improved their self‐efficacy by participating in clinical supervision through increased levels of self‐awareness and being involved in the provision of improved standards of care.

#### 3.4.13 Staff Retention

One included study reported on the effect of clinical supervision on staff retention (Robinson, Murrells, and Smith 2005). Interestingly, Robinson, Murrells, and Smith (2005) reported that those who were dissatisfied with the quality and frequency of their clinical supervision were more likely to view themselves continuing as a nurse at both 5 and 10 years into the future.

#### 3.4.14 Sick Leave, Time for Clinical Supervision and Organisational Acceptance

None of the included studies reported on the workplace outcomes of professional identity, sick leave, time for clinical supervision or organisation acceptance for mental health nurses participating in clinical supervision.

## 4 Discussion

The aim of this scoping review was to analyse the available evidence for workforce outcomes relating to clinical supervision for mental health nurses and identify any gaps within the currently available literature. An overarching finding arising from this review is that clinical supervision as an area of research is limited due to a lack of consensus around core concepts. As a result, evaluation is marked by inconsistencies/variations/lack of consensus in (1) what clinical supervision is; (2) what best practice clinical supervision is; and (3) what training is required for clinical supervision, among others (Sharrock et al. 2019). This finding aligns with a recent scoping review of clinical supervision within nursing broadly, noting that there are inconsistencies in clinical supervision definition both across and within studies (Ryu et al. 2024). These inconsistencies create challenges in synthesising results and making recommendations for best‐practice clinical supervision.

Despite this limitation, our review found clinical supervision is often cited as an approach to improving both personal and system/structural workforce outcomes associated with mental health nurses. However, the complexities of the work mental health nurses do, and the environment within which they do it, make it difficult to effectively define and evaluate the impact of clinical supervision. Most of the results included in this scoping review showed a positive association between clinical supervision and the reported individual and system outcomes for mental health nurses. However, there were some conflicts within the literature, particularly within competence, workplace culture, job satisfaction and burnout. Given the complex nature of workplace environments, it can be difficult to isolate causative factors when evaluating interventions. The majority of research in this field showing positive outcomes following clinical supervision is a reassuring finding of this review, which builds upon and extends a previous literature review examining workforce outcomes in other health professionals (Martin et al. 2021).

Evaluation research for clinical supervisions' impact on workforce outcomes for mental health nurses is mostly comprised of qualitative research or participants self‐identifying outcomes at a single point in time. When considering the different levels of evaluation evidence for interventions, evidence arising from cross‐sectional research is often considered below evaluations with longitudinal and/or randomised designs (Wallace et al. 2022). Additionally, there is also a possibility that individuals who already view clinical supervision favourably are more likely to participate in research evaluating it increasing the likelihood of self‐selection bias (Kaźmierczak et al. 2023). This has the potential to introduce optimism or familiarity biases to evaluation research, where participants overstate the positive outcomes of an intervention (Greul, Schweisfurth, and Raasch 2023). Despite these limitations, such studies can be of considerable value and are important. Priority should be given to more robust research evaluating clinical supervision with mental health nurses to support the broad findings identified in this review. Further robust evaluation of different types of clinical supervision will help to build consensus on best practice clinical supervision, providing a framework for those undertaking and providing clinical supervision to get the best outcomes for the workforce and the clients in their care. Reassuringly, randomised control trials in other healthcare disciplines have found positive workforce outcomes associated with clinical supervision (Osiurak et al. 2024), and welcome upcoming trials (Catling et al. 2022).

Studies in this scoping review rarely published details of the supervision participants in the study received, including length and frequency of sessions, the model being used, individual or group supervision and whether sessions were face‐to‐face or conducted virtually. These details were either not reported at all, or participants were receiving various types of supervision, and the results were reported such that the outcomes could not be delineated to different types of supervision. Given this, caution should be applied when attempting to attribute the results of this scoping review to specific clinical supervision contexts. It is recommended that future research evaluating clinical supervision for mental health nurses is developed and reported in such a way that outcomes of clinical supervision can be attributed to the characteristics of the clinical supervision received by participants.

## 5 Conclusion

In the current climate, initiatives are needed to support the mental health nursing workforce to stay in their jobs and provide the best quality care to clients. Despite limitations in the evidence base noted in this review, clinical supervision can offer an opportunity to provide workplace support to individuals to improve their knowledge, skills and satisfaction within a workplace. Further primary evaluation research will be needed to strengthen the evidence base and provide clarity around best practice clinical supervision.

### Relevance for Clinical Practice

It is the view of the authors that, when combined with other worker support, advocating for clinical supervision for mental health nurses is worthwhile. Based on the findings and limitations of this scoping review, there are some potential recommendations for how mental health nurses can be supported within workplaces with respect to clinical supervision. For other professions in mental healthcare provision (e.g. social workers), clinical supervision is an established part of their professional identity and a mandatory standard for accreditation (Long et al. 2024; Australian Association of Social Workers 2023). We would recommend that clinical supervision be made readily available and encouraged in the workplace for all mental health nurses to individually and voluntarily organise and accept. However, we would be cautious of recommending that organisations create policies that require clinical supervision to be made mandatory for or enforced on all mental health nurses. The reasoning behind this is threefold. First, mandatory supervision for people who are indifferent or hostile to the process undermines the benefits and voluntary nature of clinical supervision. Second, implementing clinical supervision is not without risk of supervisor–supervisee misalliance and is dependent on strong trust between the supervisor and supervisee(s). Mandating this process for people without autonomy and choice of supervisee/supervisor, and without forming a strong bond, could have adverse outcomes for supervisors, supervisees and the workplace. Finally, mandating clinical supervision without first addressing individual workloads can place an extra burden on time‐poor staff, resulting in greater stress, poorer care and negative workforce outcomes. These scenarios are counterintuitive to the workforce benefits that clinical supervision is supposed to achieve. Instead, we recommend that workplaces provide space, resources and encouragement for all mental health nurses to engage in clinical supervision. For inpatient units and community teams, adapting workload support models that allow non‐nursing staff (e.g. allied health) to cover the roles of mental health nurses to give them protected time to focus solely on clinical supervision without compromising client safety. This will ensure that clinical supervision is made available to those who want it and encourage a culture that values staff engaging with clinical supervision.

## Author Contributions

All authors were involved in the conceptualisation of this research. J.M. prepared the manuscript, with reviewing and editing provided by K.R. and N.P. All authors approved the final version of the manuscript prior to submission.

## Conflicts of Interest

The authors declare no conflicts of interest.

## Data Availability

Data sharing is not applicable to this article as no new data were created or analyzed in this study.

## Appendix 1 Search strategy developed for MEDLINE

### 1.1

**Table:**

#	Query
1	Psychiatric nursing/
2	exp Mental Disorders/nu [Nursing]
3	exp mental health/ and exp. nurses/
4	((Mental health or psychiatric or psychosocial) adj3 nurs*).ti, ab, kf.
5	or/1–4
6	(Clinical supervision or professional supervision or peer supervision).ti, ab, kf.
7	5 and 6
